# Sensing Microorganisms Using Rapid Detection Methods: Supramolecular Approaches

**DOI:** 10.3390/bios15030130

**Published:** 2025-02-21

**Authors:** Hiya Lahiri, Kingshuk Basu

**Affiliations:** Department of Biological Chemistry, The Alexander Silberman Institute of Life Sciences, The Hebrew University of Jerusalem, Edmond J. Safra Campus, Jerusalem 9190401, Israel

**Keywords:** biosensing, pathogen, bacteria, virus, fungus, supramolecular sensing, rapid pathogenic detection

## Abstract

Supramolecular chemistry relies on the dynamic association/dissociation of molecules through non-covalent interactions. These interactions of a self-assembled system can be strategically exploited for sensing several microorganisms. Moreover, supramolecular systems can also be combined with other functional components like nanoparticles, self-assembled monolayers, and microarray systems to produce multicomponent sensors with higher sensitivity and lower detection time. In this review, we will discuss how cutting-edge supramolecular chemistry has enabled scientists to develop microbial biosensors with high reliability and rapid detection time. Moreover, they produce high-throughput operations, real-time monitoring, extensive operation platforms, and cost-effective production. This review can serve as a conceptual background for understanding state-of-the-art rapid detection methods of microbial biosensing.

## 1. Introduction

Supramolecular chemistry, a relatively new field of scientific research, has emerged from a cumulative understanding of the complex biological world. Forces that hold biomacromolecules have supplied a huge conceptual background of weak chemical bonding in the last century. The discoveries of the DNA double helix by Watson and Crick [1], protein structures by Pauling [2], and the lipidic model of the plasma membrane by Singer and Nicolson [3] have expanded the understanding of chemical science to the world of complex molecular assembly. The key advantage of molecular assemblies is that they are dynamic and formed by weak chemical forces such as hydrogen bonding, Coulombic interactions, π–π, ionic–π, hydrophobic, and van der Waals interactions. Supramolecular chemistry relies on these forces [4]. Designing molecules with functional groups providing one or many of the above-stated interactions can produce self- or co-assembled structural patterns with functional outputs. Sometimes these assemblies respond quickly by local stimuli, which in turn can be utilized in several strategic ways [5]. One such strategic use is sensing, where the molecule under consideration perturbs the physical/chemical state of the assemblies, which in turn get reflected in the macroscopic observables of the supramolecular adducts [6]. Analytes may vary in nature; they can be toxic metal ions, environmental pollutants, industrial hazards, or pathological samples. Depending on the chemical nature of the analyte, strategies of sensing differ remarkably. In this review, supramolecular strategies for rapid sensing of microorganisms have been discussed with a conceptual discussion on how recent advancements in supramolecular chemistry and its integration with other scientific disciplines have produced a state-of-the-art sensing platform for microbial sensing.

**Figure 1:**
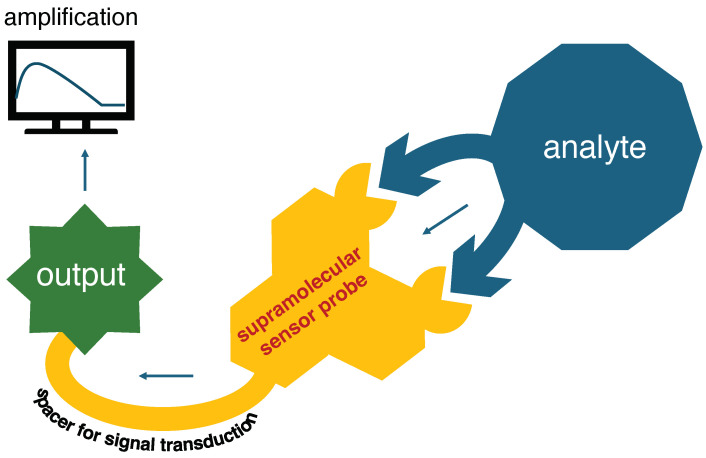
Schematic representation of supramolecular sensing highlighting components with proper functionality.

Sensing microorganisms is an essential part of biosensing due to its diagnostic importance in clinical practice. However, the detection should be fast enough and accurate at the same time to make the treatment effective. Several conventional methods have been developed and adapted by clinicians and biomedical professionals so far to identify pathogenic contaminations in pathological samples. These processes include the isolation and culture of pathogenic strains, followed by cumbersome serological and biochemical methods. Furthermore, most of them are time-consuming and often produce false assessments [7]. For example, the conventional detection of a pathogenic strain using polymerase chain reaction (PCR) techniques often produces false-positive or false-negative results [8] due to contaminations of amplified sequences [9]. On the other hand, colony counting or flow cytometry techniques lack intricate details of cell architectures [7]. For some serious ailments like multi-drug-resistant infections, these types of loopholes may turn fatal [10]. Supramolecular sensing relies on a precise interaction between an analyte and an analyst. This precision deals with the proper positioning of functional groups around a supramolecular synthon. A binding event with this synthon should exert a considerable perturbation in the molecular assembly and should produce measurable results. Figure 1 shows a schematic representation of such precise interaction and signal production. Along with precision, rapid sensing is also a key requirement for pathogen sensing. Supramolecular interactions are dynamic and fast; therefore, they produce perceivable effects within a stipulated time. Many of the supramolecular sensor probes rely on a spectroscopic shift upon pathogen binding, and hence, they show rapid spectral changeover in a short timescale [6,11].

## 2. Importance of Rapid Sensing of Microorganisms

Sensing pathogenic microorganisms is very important due to the current enhanced demand in the healthcare sector. According to WHO Priority Pathogen List 2024, 19 high-priority pathogenic bacteria have emerged as potential threats in the healthcare sector [12]. On top of that, global healthcare has gone through a huge pressure from pandemic management due to the recent COVID-19 infection. Therefore, the current sustenance of healthcare relies on the rapid detection of pathogens in both human and animal samples. To address this, the “One Health” initiative has emerged as holistic care for pathogen management and pandemic care among both human and animal populations [13]. This initiative critically asses the interconnectedness of pathogenic infection between human and animal mass. The rapid transduction of diseases by transboundary outbreaks can only be controlled by rapid and reliable diagnostics [14]. Infection management cannot be done without fast diagnostics and is difficult to achieve by traditional genotypic or phenotypic analysis. On that account, only rapid biosensing methods hold the prospect of effective healthcare. The economic perspective and long-term well-being of treatment are also dependent on rapid diagnostics. Recent reports have shown the economic benefits of fast diagnosis in a point-of-care technique [15]. Moreover, it also reduces the use of antibiotics and lowers the risk of antibiotic resistance [16]. The pandemics in the last decade, including Ebola in 2014–2016, Zika, and COVID-19, have propelled huge research and development costs in rapid diagnostic techniques to reduce healthcare costs and improve outbreak response [17].

## 3. Multivalency of Supramolecular Sensing

Supramolecular probes are super-efficient in sensing biomolecules. Inspired by nature, several supramolecular probes have been designed and successfully utilized for detecting biomolecules. Positively charged dye molecules can stack on DNA strands depending on the sequence specificity to produce a stacked structure with fluorescence shift [18]. The binding of proteins with a substrate is a key condition for several biological interactions present in nature. Acquiring knowledge from that, supramolecular chemists have designed several sensing probes with peptides and polymers (will be discussed later in detail). Additionally, using several spectroscopic and microscopic tools, supramolecular chemistry has grown into a multivalent research field to serve the purpose of everyday life [19]. Therefore, a discussion of the conceptual background of the field is necessary from the view of different key supramolecular materials reported in the literature.

### 3.1. Polymer Conjugated Supramolecular Materials for Microbial Detection

**Figure 2:**
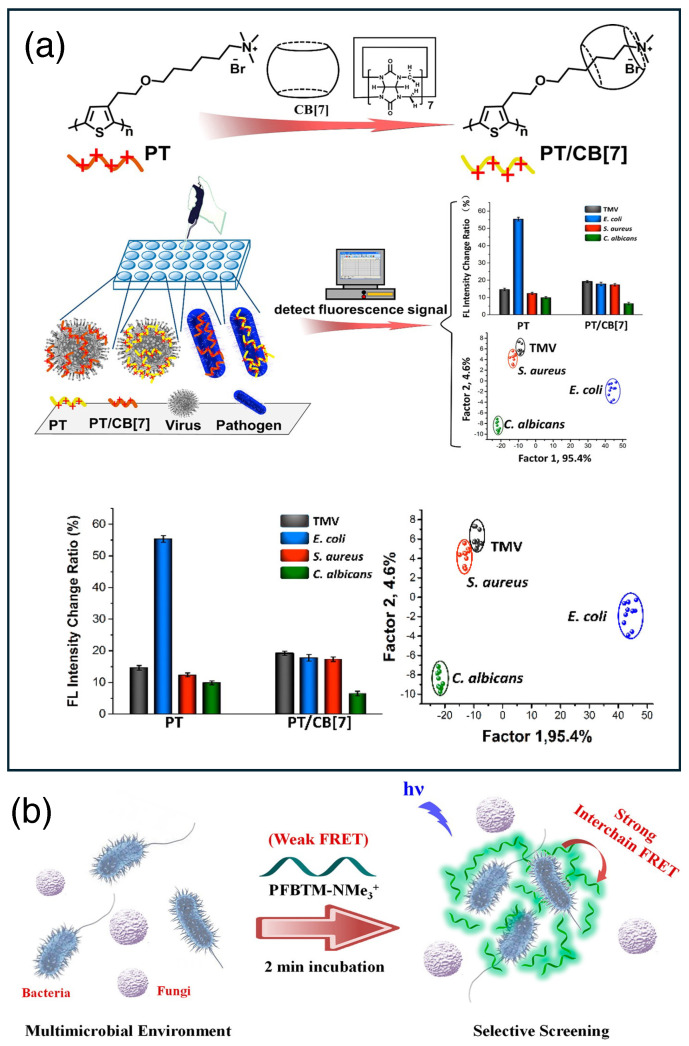
(**a**) Host-guest-based pathogen detection platform. A cationic conjugated fluorescent polymer acts as a sensor probe for pathogens, and from the fluorescent output, the nature of the pathogen is detected [23] (reproduced with permission from the American Chemical Society). (**b**) FRET-based probe for pathogen detection where binding triggers strong interchain FRET [25] (reproduced with permission from the American Chemical Society).

The essence of supramolecular chemistry lies in the rapid change in molecular assembly or interaction upon perturbation and its manifestation in macroscopic observables. Polymer-based supramolecular chemistry is one of the most efficient ways to fabricate biosensing platforms. These materials are a unique combination of supramolecular and polymer chemistry and formed by joining molecular units through both covalent and non-covalent interactions. Polymer-conjugated supramolecular materials are found to be very effective in detecting microorganisms and relevant pathogens. Electrochromism is a nice observable phenomenon recently utilized by Wu et al. to discriminate Gram-positive (G+) and Gram-negative (G−) bacterial strains. They used an electrochromic copolymer, poly(3,4-propylenedioxythiophen-alt-3,4-ethylenedioxythiophene) (PPE), for the visible sensing of different bacterial strains of *Staphylococcus aureus* resistant and responsive to antibiotics within 2.5 h [20]. Tethering antibacterial drugs to a polymeric scaffold can produce rapid sensing platforms. Xu and co-workers attached Vancomycin to a polythiophene derivative containing reactive pentafluorophenyl found to be effective in the rapid detection of G+ bacterial strains. Vancomycin binds and kills G+ bacterial strains; therefore, the drug-appended polymer selectively binds with G+ bacterial strains and shows a change in the fluorescence of the resulting polymer-bound bacteria [21]. Wang and co-workers invented a method that is much faster (2 h) than traditional methods to detect pathogens and also does not need any specific biomarkers for labeling. In this work, they used a cationic poly(fluorene-co-phenylene) derivative (PFP-NMe^3+^) that forms a reversible supramolecular complex with cucurbit[7]uril (CB[7]); amantadine (AD) disassembled it and released PFP-NMe^3+^. When pathogens bind with the complex, it interacts differently as the hydrophobic interaction of PFP-NMe^3+^ changes before and after assembly. This system serves as an in situ optical sensor that can rapidly detect and discriminate among various pathogens, including Gram-negative and Gram-positive bacteria and fungi [22]. In a recent publication, they showed how a supramolecular polymer conjugate can be used to discriminate viruses and microbes. They developed a strategy to discriminate viruses and microbes by utilizing supramolecular chemistry with a cationic polythiophene derivative (PT) and cucurbit[7]uril (CB[7]). This approach can differentiate between viruses and microbes with the changes in polymer fluorescence intensity, which is caused by the different interaction patterns with PT and PT/CB[7] complexes. They also added linear discriminant analysis (LDA) to enhance the discrimination efficiency (Figure 2a) [23]. The interaction of a polymeric quaternary ammonium group with a bacterial surface has been recently addressed by He et al. as a joint effect of a hydrophobic effect and zeta potential [24]. This type of quaternary ammonium group appended polymer with a Förster Resonance Energy Transfer (FRET) couple can sense Gram-negative bacterial strains with high efficacy. After binding with bacteria, the polymer gets aggregated, which is effective in discriminating bacterial and fungal strains (Figure 2b) [25]. The photoelectrochemistry of conjugated polymers with suitable charge components is effective in the design of a sensing platform. Zhou et al. utilized the photoelectrochemistry of a positively charged poly(phenylene vinylene) derivative (PPV). The positive charge on the derivative helps it to bind the negatively charged electrode surface and to produce a photo-induced electron transfer process. Now, the addition of bacterial strains with a negatively charged membrane forms adducts with a positively charged PPV-coated electrode surface, which eventually hinders the photoelectron transfer and acts as a sensing platform [26].

### 3.2. Peptide-Based Pathogen Sensing

**Figure 3:**
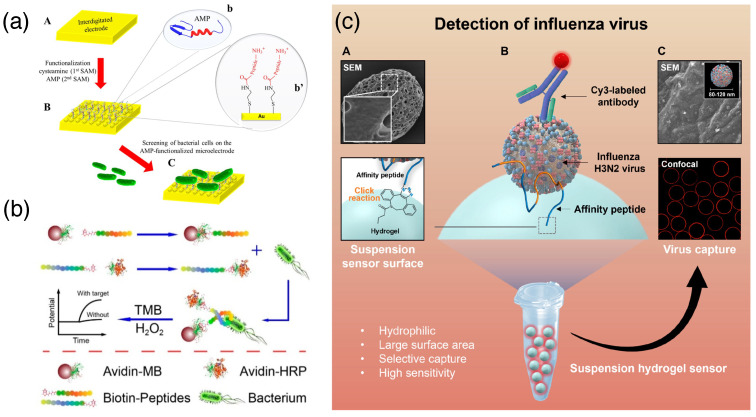
(**a**) Antimicrobial peptide (AMP) for the detection of bacterial strains: (**A**). integrating microelectrode, (**B**). AMP modified surface, and (**C**). bacterial binding on surface [29] (reproduced with permission from the American Chemical Society); (**b**) sandwich assay for antimicrobial peptide with magnetic bead and redox detection probe for the potentiometric detection of *Listeria monocytogenes* (reproduced with permission from the American Chemical Society) [30]; and (**c**) peptide-hydrogel sensor for Influenza virus, using affinity peptide conjugated through click chemistry and antibody tagged with fluorescent probe ((**A**,**C**) shows scanning electron microscopic images of hydrogel and virus-bound hydrogel respectively. (**B**) represents the sensing mechanism of the hydrogel by using a combination of affinity peptide and labeled antibody) [34] (reproduced with permission from the American Chemical Society).

Peptides are excellent supramolecular probes for various functional uses. The advantage of peptide probes lies in their easy functionalization using various combinations of component amino acids, relatively easy and cost-efficient fabrication, and inherent robustness [27]. Antimicrobial peptides are effective probes for such detection. They can easily target bacterial membranes with their cationic charge and hydrophobic residues. Several types of secondary structures are observed in antimicrobial peptides, such as α–helix, β–sheet, and extended structures [28]. An antibacterial peptide, leucocin A, has been covalently immobilized on gold microelectrodes via the C-terminal −COOH and the amine end of a thiol linker present on a Au surface. This Au-electrode array was subjected to an impedimetric study to detect Gram-positive bacterial strains (Figure 3a). This peptide can selectively sense *Listeria monocytogenes* among other G+ strains from food samples with a detection limit of 103 cfu/mL [29]. A sandwich-type assay of an anti-*Listeria* peptide has been fabricated by splitting it into two parts. The capture probe was attached to magnetic beads, and the detection fragment was labeled with horseradish peroxidase for catalyzing the oxidation of 3,3’,5,5’-tetramethylbenzidine with H_2_O_2_. Using this conjugate and polymeric membrane ion-selective electrode (ISE), the detection of 3,3’,5,5’-tetramethylbenzidine oxidation events has been performed potentiometrically as a marker of *Listeria* strains in pathological samples (Figure 3b) [30]. Clamp peptides are antibody-mimicking short peptides with a high affinity toward viral particles. Recently, Mascini et al. designed a clamp peptide with two detection arms and a hinge arm to bind the envelop protein of Zika virus. They used colorimetric detection for the study with a detection limit of 10^5^ copies/mL [31]. Park and co-workers, in their recent study, showed that a specific peptide, EHDRMHAYYLTR (R3#10), can serve as a biomarker by targeting the dengue virus type 2 NS1 protein (DENV2 NS1) via phage display and with the help of cyclic voltammetry (CV) and electrochemical impedance spectroscopy (EIS) [32]. Sajjanar et al. introduced a conjugated system of peptide–gold nanoparticles (AuNPs) for sensing viruses by fast and selective visual-based sensing. Cysteinylated virus-specific peptides were used to functionalize citrate-stabilized AuNPs, and this conjugate system formed a complex in the presence of viruses by forming an aggregate. This aggregation is observed in terms of color change by the naked eye or by UV–Vis spectrophotometry. This method is PCR amplification independent and has a detection limit of 0.125 HA units of the virus [33]. Park and co-workers introduced a rapid and accurate novel biosensor to detect pandemic flu viruses. With this method, Influenza A virus H3N2 was detected with the help of affinity peptide-immobilized hydrogel microspheres. This hydrogel microsphere binds with a specific virus (H3N2), and the subsequent addition of fluorescent-tagged haemagglutinin-specific antibody shows fluorescence in the solid phase detector with a detection limit of 1.887 PFU/mL (Figure 3c) [34].

### 3.3. Fluorescent Probes for Pathogen Detection

**Figure 4:**
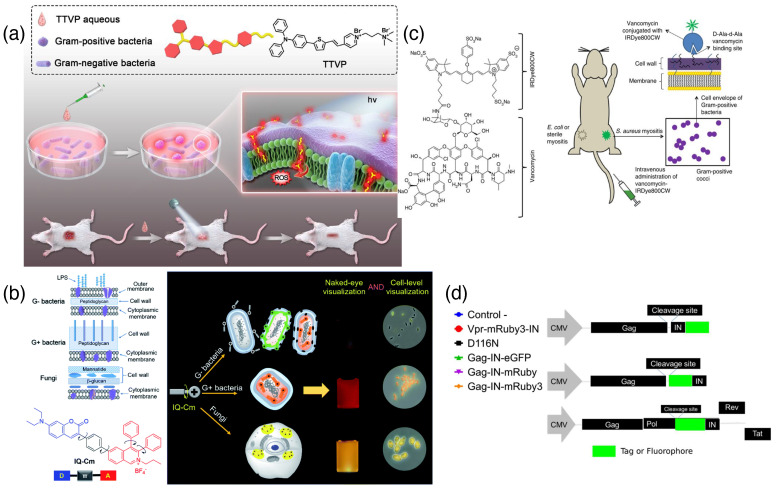
(**a**) Infra-red emissive AIEgen TTPV dye molecule (upper inset) for the detection and killing of Gram-positive bacteria [37] (reproduced with permission from Elsevier); (**b**) chemical structure of G+ and G– bacterial and fungal envelops along with AIEgen dye molecule with donor–π–acceptor (left panel). Naked eye detection of bacterial and fungal strains using the IQ–Cm molecule (adapted from reference [38]); (**c**) structure of Vancomycin conjugated 800CW dye (left panel), mode of detection using the dye-antibiotic conjugate (adapted from reference [42]); (**d**) schematic depiction of recognition proteins conjugated with fluorescent tag for imaging HIV particles (adapted from reference [44]).

Changes in the fluorescence of supramolecular binding probes effectively detect pathogenic samples rapidly and efficiently. Fluorescent detection techniques are endowed with easy handling; therefore, less efficient hands are required for these techniques [35]. Aggregation-induced emission (AIE) is a unique feature of fluorescent probes, where the aggregation of fluorophores triggers the radiative deactivation of the photon-fluorophore complex [36]. Collectively, these detection agents are called AIEgens. They have the advantage of excellent sensitivity and are useful for the detection and imaging of bacterial strains. TTVP (Figure 4a) is a nice example of an organic luminogen that emits in the near-infrared region (NIR) with the dual application of Gram-positive bacteria discrimination and photodynamic therapy. Moreover, this can discriminate G+ bacterial strains with a washing-free ultrafast staining procedure [37]. In AIEgen molecules, the common principle rests in the aggregation-induced restriction of the molecular rotation of fluorophores. Recently, Zhou et al. demonstrated a coumarin-containing dye molecule, IQ–Cm, which has a donor–π–acceptor arrangement (Figure 4b) and can selectively bind Gram-positive bacteria. This dye is efficient in the diagnosis of urinary tract infection (UTI) in clinical samples (Figure 4b) [38]. Tetraphenylethylene (TPE) is an interesting molecule where the rotation of phenyl rings gets restricted upon aggregation. The hydrophobicity of the dye molecule was tailored by putting different hydrophobic groups in the quaternary ammonium group. Additionally, alkoxy groups were introduced to increase the hydrophilicity of the dye molecule to make the detection better. Linear discriminant analysis (LDA) was made to discriminate different bacterial strains using these dye molecules [39]. Putting proper labels on AIEgen can make an appropriate platform for the detection of bacterial infection visible at the cellular level. Recently, Liu and co-workers tagged tetraphenylethylene (TPE) moiety with a casp-1 cleavable protein to image bacterial infection under the microscope. Casp-1 is produced upon bacterial infection and cleaves the NEAYVHDAP sequence of peptide-tagged TPE to trigger AIE of TPE [40].

One of the efficient ways to target pathogens with fluorescent probes is to tag the probe with a proper biomarker that can target the pathogen surface or component rapidly [41]. Peptidoglycans (PGs) are good targets in bacterial and fungal cell walls. Vancomycin (Van) is a known drug that targets the (D)Ala-(D)Ala moiety of G+ bacterial lipid II; therefore, tagging fluorophores with Van can be a nice trick to image and detect bacterial strains. Oosten et al. tagged an infrared-emitting dye 800CW (Figure 4c) with 775 nm absorption and 792 nm emission values. This can be useful in the real-time imaging of bacterial infection in a mouse myositis model and a human post-mortem implant model [42]. Lipopolysaccharides (LPS) are another useful target for bacterial sensing. AIEgen probes that can electrostatically get attached to the LPS layer are also efficient platforms for sensing bacterial strains [43].

Fluorescent tags on viral surrogate proteins have been found to be effective in imaging live viral strains. Recently, Mamede et al. reported a method for tagging the integrase protein of human immunodeficiency virus 1 (HIV-1) with another fluorescent protein. This tag is effective in identifying viral complexes of the HIV-1 infection cycle (Figure 4d) [44]. To make the sensing rapid and less complex, the recent incorporation of synthetic dyes and quantum dots has been proven very effective [45]. An indicator displacement assay is another key assay used in supramolecular fluorescent sensing. Recently, a fungal mycotoxin, ochratoxin A (OTA), has been detected using albumin as a host and a flavonoid fluorescent indicator as the guest. The displacement event has been detected by a conventional fluorescence outcome with a sensitivity of 0.39 ppb and a 5 s detection time [46].

### 3.4. Nanomaterial Conjugates

An extraordinary way to fabricate a supramolecular sensing platform is to extend the concept of supramolecular chemistry to the regime of nanomaterials. Conjugating fluorescent polymers to silver nanoparticles endowed with surface plasmon resonance provides the portability of the assembly and increases its effectiveness in bacterial sensing. These materials have been proven to have anti-microbial effects [47]. Recently, Li et al. reported Ag-nanotriangles (AgNTs) with differential adsorption towards Gram-positive and Gram-negative bacterial strains. They used different structures as colorimetric probes for different bacterial strains by adsorbing one kind of bacteria (G+ or G−) and leaving the other free to react with the AgNTs to produce colorimetric output [48]. Nobel metal nanoparticles with few atoms show good fluorescence properties. Atomically precise gold nanocluster Au_102_(p−mercaptobenzoic acid)44 functionalized with maleimide linkers has been targeted to the cysteines of viral capsid proteins. This method has been found useful for the microscopic visualization of the viral particles, including their entry mechanism [49].

**Figure 5:**
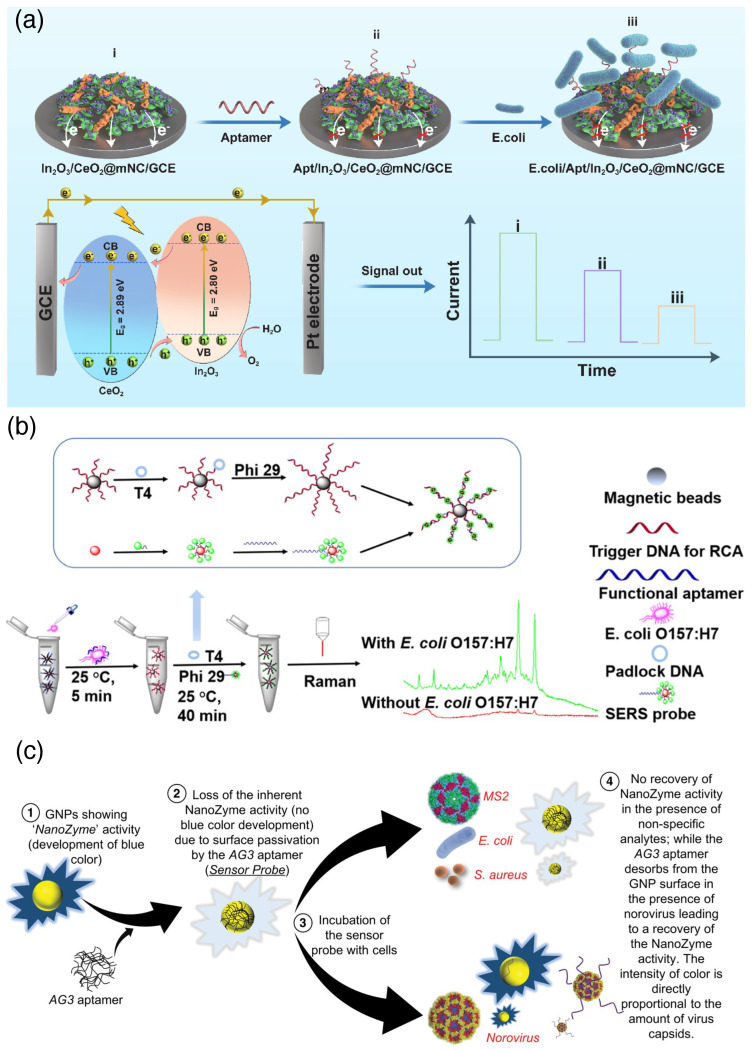
(**a**) Aptasensing of *E. coli* using In_2_O_3_/CeO_2_/aptamer hybrid using photoelectrochemical assay [53] (reproduced with permission from Elsevier); (**b**) Au-nsanoparticle-based surface-enhanced Raman scattering (SERS) adaptive sensor coupled with rolling cycle amplification (RCA) for the detection of *E. coli* [54] (reproduced with permission from the American Chemical Society); (**c**) nanoenzyme activity of Au-nanoparticle (AuNP) used for bacterial detection where AG3 aptamers passivate the AuNP surface, and the catalytic activity is lost. Upon the addition of Norovirus, the aptamer is chunked out, and the catalytic activity of AuNP is regained [55] (reproduced with permission from the American Chemical Society).

Aptamers are fragments of DNA or RNA with functional recognition sites that can bind biomolecules depending on the primary nucleotide sequences. Aptamers attached to nanomaterials make excellent sensing platforms with a rapid sensibility to pathogens [50,51,52]. In_2_O_3_/CeO_2_-nanocrystals are endowed with a nice electrochemical response, and attaching aptamers to them has produced an efficient *E. coli* sensor (Figure 5a) with a sensing limit of 1.12 CFU mL^-1^. This is lower than most of the *E. coli* sensors reported so far [53]. On the other hand, Au-nanoparticles are endowed with surface-enhanced Raman scattering (SERS). Attaching aptamers to them brings the pathogen sensing in the SERS window and makes the detection more precise and less time-consuming. Recently, Zhang and co-workers combined SERS-based sensing with the rolling cycle amplification (RCA) process of DNA to bring the *E. coli* signal within the Raman spectral range. This process is one pot where DNA aptamers, attached with magnetic beads, undergo RCA and get hybridized with sensor probes tagged with AuNPs and show Raman peaks as a marker of pathogens (Figure 5b) [54]. Gold nanoparticles also have enzymatic activity; combining that with aptosensing has produced an excellent Murine Norovirus (MNV) detection tool. Bansal and co-workers have combined AuNPs and an MNV AG3 aptamer to develop a colorimetric sensor for a rapid and highly specific detection of Norovirus in 100 μL of sample in 10 min. AuNPs with nanoenzyme activity show a blue color in a colorimetric assay. MNV AG3 aptamer molecules get adsorbed on a AuNP surface to passivate the enzyme activity, so no blue color is seen. Virus-containing samples bind with the viral particles to regain the nanoenzyme activity, so the solution’s blue color recovers (Figure 5c) [55].

### 3.5. Label-Free Methods

**Figure 6:**
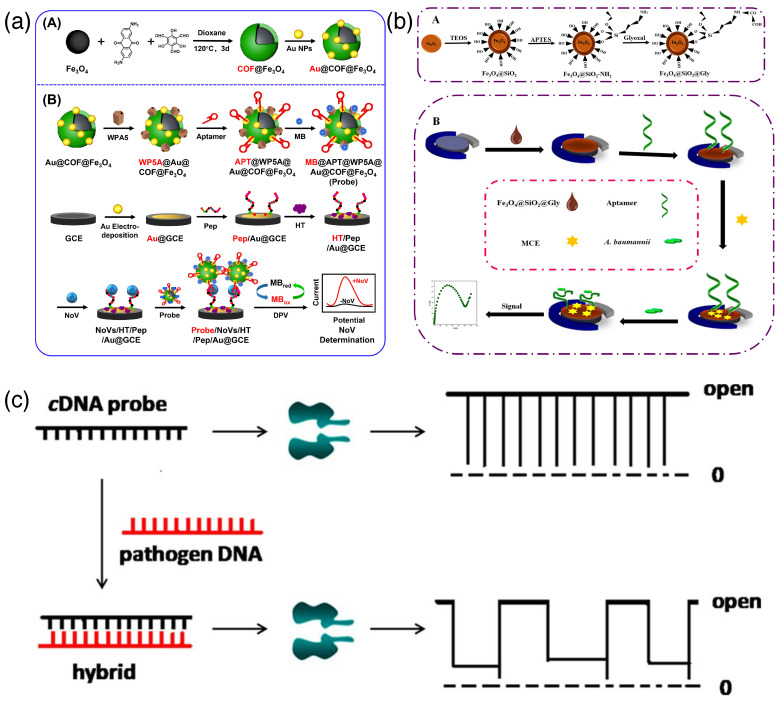
(**a**) Hybrid magnetic nanoparticle functionalized with aptamers for the detection of a human Norovirus sample using electrochemical assays. Subfigure (**A**): synthetic scheme of Au@COF@Fe_3_O_4_; Subfigure (**B**): Sensing mechanism of Au@COF@Fe_3_O_4_ appended with aptamer [63] (reproduced with permission from Elsevier); (**b**) label-free nanohybrid aptasensor for the detection of *Acinetobacter baumannii* bacteria by impedance measurements (Subfigure (**A**,**B**) shows synthetic scheme of the nanoparticle fabrication and sensing mechanism respectively) [64] (reproduced with permission from Elsevier); and (**c**) detection scheme of an Anthrax viral genome using nanopore assembly, where the hybridization of an aptamer probe changes the resultant current pattern (adapted from reference [66]).

Label-free detection methods are free from labeling and can detect pathogens without tampering with their native states. Label-free methods detect the analyte samples in their purest state without putting any tag or label on them. Methods involved in such analyses are very sensitive and precise. One of the most celebrated tools for label-free biosensing is the surface plasmon resonance technique. In this method, the incident light on a metal surface interacts with the evanescent electron wave of the surface. At a particular incident angle where the incident photons get in phase with the electron wave, a huge absorption of energy takes place. Now, any change in the surface property leads to a change in the value of this incident angle, and we can see that change as a marker of sensing [56]. This key method has had huge development in the last few decades to form a state-of-the-art platform for biosensing [57,58,59]. Moreover, in most cases, it is integrated with signal amplification devices with a remarkably low response time [57]. For example, a common strategy is to immobilize a solid surface with an antibody specific to a pathogen. Upon binding that particular pathogen to the solid surface decorated with an antibody, an optical signal is sensed or imaged to confirm the binding event [58]. This surface binding method has much to do with the supramolecular chemistry of the ligand. Fungal B1 aflatoxin (AFB1) has been detected electrochemically by the supramolecular engineering of a streptavidin layer immobilizing biotinylated anti-AFB1 antibodies. The method allowed the fabrication of a printed electrode surface and made the detection as low as 5 ng/mL with a detection limit of 50 fg/mL. This range and limit are lower than many of the reported toxin detection limits available in the literature [60]. Atomic force microscope-based single molecule force spectroscopy is another example of a cutting-edge label-free detection method that works at the single-molecule level. In this technique, a microscopic tip functionalized with detector species approaches an analyte species and forms an adduct; now, the restoration force on the tip pulls back the adduct apart to a non-bonded state, and the resulting force is recorded at the single-molecule level [61]. A surface, modified with synthetic nucleic acid analogues such as locked nucleic acids (LNAs), can efficiently sense point mutation in the pathogenic genome. Gene mutations in the rpoB gene of *Mycobacterium tuberculosis* have been successfully detected by a fast and label-free sensing technique using atomic force spectroscopy employing on-surface LNA probes [62]. Hybrid nanostructures have a much higher sensing ability and are rapid in terms of detection time scale. A magnetic covalent network composed of Fe_3_O_4_, hybridized with pillararene heterosupramolecular nanocomposites and functionalized with peptides and aptamers, has been utilized efficiently for the detection of Noroviruses in 98 clinical samples (Figure 6a). Here, the accuracy was found to be higher than the conventional immunochromatographic assay without any extraction and amplification of viral nucleic acids. The detection limit was found to be 0.84 copy mL^-1^ for the human Norovirus [63]. Aptasensors can also be used in label-free detection methods. Aptamers have been immobilized on an Fe_3_O_4_@SiO_2_@Glyoxal (Gly) modified electrode, and the outcome was measured as a function of the impedance of an [Fe(CN)_6_^3-/4-^] couple (Figure 6b) [64]. Nucleic acid components of pathogens can be detected using nanopores, an elegant, label-free, and highly efficient method used in clinical practice nowadays. The essence of this process lies in its high specificity at the single-molecule level with the time of response. Nanopores are fabricated by the supramolecular organization of biomolecules, supported on 2D materials such as graphene, silicon, and quartz [65]. Anthrax lethal toxin has been detected using a protein nanopore attached with complementary DNA (cDNA) as the probe molecule. cDNA alone can pass the nanopore easily with a particular type of current response curve. On the other hand, upon hybridization with an Anthrax toxin-DNA sample, a dsDNA molecule is formed, and the resulting molecule faces difficulty in passing the pore. Now, only upon unzipping, it can pass the nanopore, resulting in a different pattern of current through the nanopore (Figure 6c) [66].

### 3.6. Lab on a Chip

**Figure 7:**
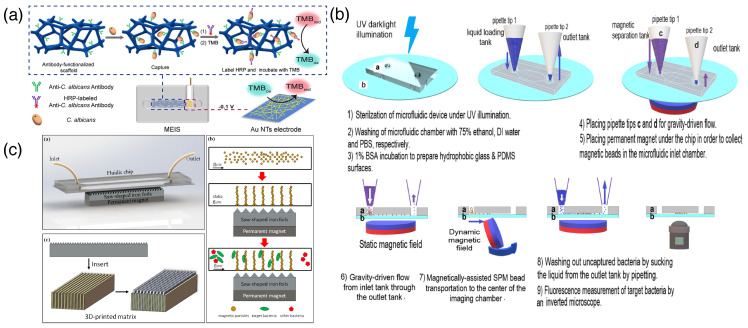
(**a**) Lab-on-a-chip detection method by using antibody-functionalized scaffold to detect *Candida albicans* [71] (reproduced with permission from the American Chemical Society); (**b**) microfluidic device for accumulating and imaging a trace amount of bacterial strains using antibody-modified magnetic beads [73] (reproduced with permission from Elsevier); (**c**) fluidic chip for assembly of magnetic beads enables bacterial detection (adapted from reference [74]).

Lab on a Chip (LOC) or microfluidic chip-based detection techniques is one of the most important label-free techniques. This is a miniaturized device that allows us to analyze multiple biological samples in a single compact platform. It has several applications, especially in early disease diagnostics and biosensing. The unit of lab-on-a chip setup is primarily made up of a set of microfluidic compartments that have different functions, such as fluid mixing, flow control, sample preparation, detection, and readout [67]. LOC is fabricated using the photolithography principle. These devices are mainly fabricated in paper or polymeric materials; as a base component, ceramic, glass, silicon, or metal can be used. Different disposition methods can be used, such as injection molding, embossing, and electroplating [68]. The major advantages of LOC in biosensing are as follows: It needs a very small amount of analytes, small analysis time, less wastage of chemicals, and comparatively lower expenses for reagents. Additionally, microfluidic systems have high throughput and show higher versatility. There are some disadvantages of LOC, which are worth mentioning, such as its complexity and its effectiveness at small-scale experiments. Usually, these are not suitable for a larger scale. A few factors, such as surface tension and clogging of a microfluidic channel, can hamper the overall efficiency of the LOC apparatus. Microfluidic chip-based or lab-on-a-chip-based methods are comparatively new, promising, and fast in monitoring pathogens like bacteria, viruses, and other pathogens that cause major environmental and health threats. Foudeh et al. showed how an LOC device can be used to detect pathogens for point-of-care diagnostics [69]. Hao and co-workers demonstrate how a polydimethylsiloxane (PDMS)/glass microfluidic chip with integrated bioluminescence technology can be used as a platform to detect *Escherichia coli* (*E. coli*) O157:H7. The detection limit was a $3.2\times 10^{1}$ cfu/µL to $3.2\times 10^{5}$ cfu/µL concentration of *E. coli* [70]. Recently, three-dimensional macroporous polydimethylsiloxane (PDMS) and a Au nanotube-based conjugate have been employed for the detection of *Candida albicans* in a microfluidic electrochemical integrated sensor (MEIS) device setup. The detection range was 30–3,000,000 CFU in clinical samples within 1 h (Figure 7a). This method holds a potential capability for the detection of other pathogenic strains as well [71]. A quartz crystal microbalance (QCM) chip-based device has been utilized for a device-based detection of *Brucella melitensis (B. melitensis)* in milk. Here, magnetic nanoparticles of Fe_3_O_4_ were modified with 3-aminopropyltriethoxysilane (APTES) and then grafted in a polymeric bed. The final layer of the nanoparticle was decorated with aptamers sensitive to *B. melitensis* and employed within the chip-based setup of a QCM device to analyze real samples [72]. Supermagnetic beads, modified with streptavidin, were subjected to a microfluidic device to capture an ultra-low concentration of *Escherichia coli* (*E. coli*) and concentrate it for microscopic imaging (Figure 7b). This method can detect bacterial strains within 1 h at a concentration of 1 PFU/mL. Moreover, the methodology is very simple and does not need expert hands [73]. Magnetic assemblies of antibody-modified magnetic nanoparticles induced with a saw-shaped iron foil have also been found to be useful for immunoseparation and the detection of bacterial strains with high efficiency (Figure 7c). This method can also be used for a future low-cost efficient biosensing platform for different bacterial strains [74].

### 3.7. Artificial Intelligence and Rapid Detection

**Figure 8:**
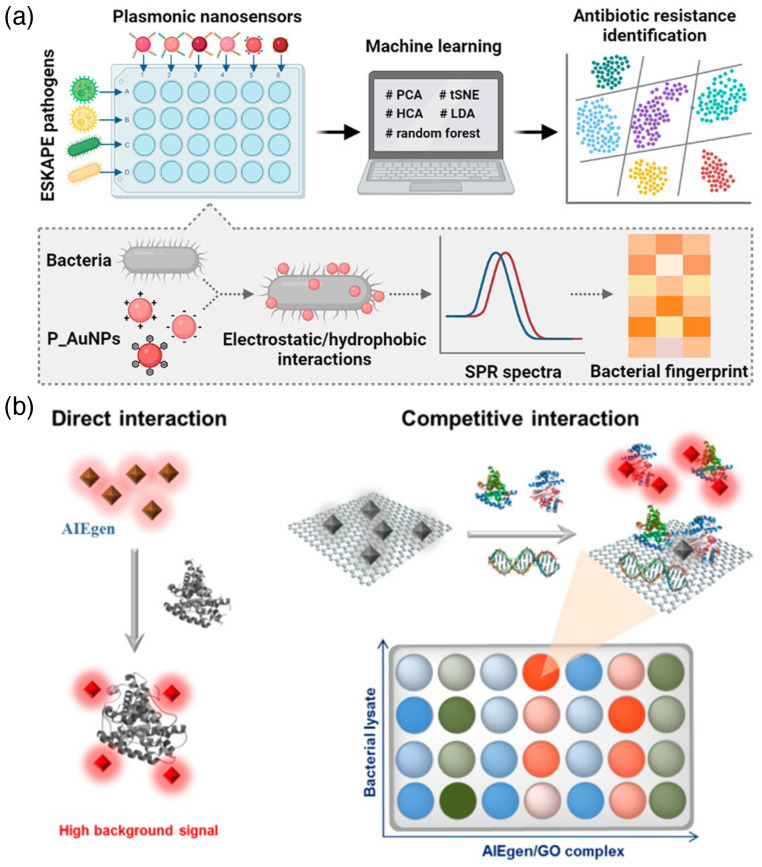
(**a**) Schematic representation of the detection platform for assaying antibiotic resistance in six bacterial strains through plasmonic nanosensors using machine learning [75] (reproduced with permission from American Chemical Society); (**b**) AIEgens-graphene oxide adducts for sensing of bacterial strains using machine learning [78] (reproduced with permission from the American Chemical Society).

Supramolecular chemistry aided with cutting-edge engineering science can produce ample amounts of sensing data. Processing them can obtain a huge leverage upon integration with data science, artificial intelligence, and machine learning. On top of that, the time of data processing and analysis can delay the detection process, and AI can manage this issue with high efficiency. Recently, Tu et al. reported a machine-learning-based platform for the detection of antibiotic resistance in six pathogenic strains, namely, *Enterococcus faecium* (*E. faecium*), *Staphylococcus aureus* (*S. aureus*), *Klebsiella pneumoniae* (*K. pneumoniae*), *Acinetobacter baumannii* (*A. baumannii*), *Pseudomonas aeruginosa* (*P. aeruginosa*), and *Enterobacter* species (ESKAPE together). The genotypical identification of these strains is cumbersome and time-consuming. Therefore, they developed peptide-conjugated Au-nanoparticle plasmonic array-based sensing, relying on the difference of surface potential and hydrocarbon binding ability of individual bacterial strains. The peptide conjugates vary in hydrophobicity and charge, and therefore, their binding affinities differ for different strains, depending on the surface charge and binding affinity of that particular strain. The surface plasmon resonance peaks of different bacterial strains on different Au-nanoparticles were collected and processed through a machine learning platform to make an antibiotic resistance identification (Figure 8a) [75]. Wang et al. also utilized a similar concept of surface hydrophobicity of the bacterial species to produce a high-throughput array based sensing with fluorescent lanthanide nanoparticles. They made a cocktail nanoprobe kit to fingerprint the physicochemical nature of the bacterial strains. With training data from known bacterial strains, unknown bacterial biofilms have been detected with high accuracy [76]. AIEgens discussed in Section 3.3 can also be employed in array-based intelligent sensing. They also have the advantage of precise fluorescent outcome, which can produce a big dataset depending on the nature of both the probe and the pathogen. Jiang and co-workers utilized TPE-AIEgens to make a fluorescent array, and based on their interaction with different bacterial strains, they made a smart statistical analysis-based detection model of pathogens present in water samples [77]. Recently, Tang and co-workers reported an elegant method for detecting a bacterial strain in an AIEgen array platform. They utilized bacterial lysis buffer for comparative binding with different dye molecules adsorbed on graphene oxide and fed the data in a statistical model to analyze and distinguish the bacterial strains (Figure 8b) [78]. This type of algorithm-based machine learning has also been recently extended for colorimetry-based Fe-single atom nanoenzymatic sensing method. This method utilizes different recognition motifs such as boric acid, antibiotics, and cetyltrimethylammonium bromide (CTAB).

## 4. Supramolecular Detection Methods: Comparative Discussion and Summary

Supramolecular chemistry, inspired by life, has been devoted to the betterment of life for a few decades. The strategic development and fabrication of devices using supramolecular chemistry have been a key step in using it in daily applications. We have discussed the current development of the supramolecular sensing of bacterial, viral, fungal, and parasitic species in terms of either material or strategic sense. Polymer or peptide-based detection deals with the material view of sensing platforms, whereas subsequent sections deal with the methodological view of the subject. Throughout the review, a few interesting facts can be summarized:

- Supramolecular sensing techniques are rapid and efficient at the same time.
- Low cost and relatively easy handling make the sensing techniques economically viable.
- Conjugation of nanomaterials with supramolecular probes further enhances the advantages of sensing.
- The integration of supramolecular nanomaterials to the device is easy and takes the process one step ahead to the clinical uses.

However, it is also important to discuss the relative sensitivity of different methods discussed in this review. In terms of methodology, fluorescence techniques have higher sensitivity than most conventional spectroscopic methods. Therefore, most of the detection methods discussed in this review are based on fluorescence outcomes of the supramolecular ligands. They are, in many cases, cheap and easy to handle [36]. The scope of fluorescent techniques is also high; e.g., other than spectroscopic sensing, fluorescence lifetime imaging is also a very important tool to evaluate the nature of sensor analyte binding [79]. However, this elegant technique also suffers a drawback. Fluorescence methods are, in most cases, not label-free, and several false-negative results are generated due to auto-fluorescence and background fluorescence [80]. Moreover, putting a label on the analyte requires a long time of sample preparation. At this point, the advantage of label-free techniques is worth mentioning [81]. These methods are, as mentioned earlier, capable of working at a single molecular resolution and therefore suffer less error [57,58,59]. However, many of these label-free methods suffer from high initial costing of infrastructures and demand trained hands for operation. Lab-on-a-chip devices are a nice manipulation of cutting-edge engineering science and supramolecular chemistry. It uses all kinds of sensing techniques, such as fluorescence, micro-mechanical transducers, and acoustic signaling devices. Moreover, the use of aptamers, enzymes, metal complexes, antigens, and many other versatile types of sensor probes has endowed it with a huge scope of application [82]. Lastly, it is very important to make some comment about the widespread application of nano-chemistry, which finds its use in all the aforementioned methodologies: as a fluorescent probe, a label-free sensing device, a sensor platform in microfluidic device, and many others.

## 5. Future Prospect

From the above discussion, it is clear that future-generation pathological sensing techniques are going to be an integration of supramolecular chemistry and their device integration. Different scientific disciplines across the world are using supramolecular strategies to develop novel devices. From the material point of view, polymer, peptide, fluorescent, and nanomaterials have progressed a lot during the last few decades. By cutting their production cost and using the combined effect of intelligent material design, based on currently available data in literature, a huge upliftment in supramolecular sensors is still possible. Integrating spectroscopic techniques such as the utilization of ultrafast spectroscopy and supramolecular biosensing can be more accurate and less cumbersome [83]. Device integration is another important feature of biosensing [4]. Making a workable platform requires proper assembly of chemical and engineering interfaces. In this review, we have discussed device development in Section 3.5 and Section 3.6. Most of them can be fabricated as clinically usable devices. References [63,64,66] shown in Figure 6 are excellent device platforms with very fast detection efficacy. On the other hand, references [71,73,74] are less conventional supramolecular approaches yet endowed with great efficiency. However, there is still more scope for several label-free methods to come into a clinically usable platform. Scientists are trying to make robust supramolecular assemblies with label-free sensing capability [84]. A combination of devices with more elegant, efficient, and robust supramolecular assemblies is therefore possible in the future. The detection of multiple organisms and differentiating them from each other more specifically (in terms of genre and nature) needs higher efficiency of the analytical tools. In this regard, supramolecular microbial biosensing has enough room for development.

## 6. Conclusions

In conclusion, it can be stated that supramolecular chemistry holds the power to detect a wide range of pathogenic strains within a very short detection time, with very high efficiency. Moreover, most of these techniques rely on easy handling and low cost, making them clinically viable in all respects. Integrating conventional molecular probes such as aptamers with nanomaterial platforms increases the efficiency of the detection platform to a much higher extent. The device integration of these nano-conjugated systems is easier and has been utilized in the fabrication of clinically viable devices. Future progress in robust supramolecular chemistry, along with their device integration, should replace time-consuming techniques with smart device-based platforms in clinics.

## Abbreviations

The following abbreviations are used in this manuscript:

AD	amantadine
AFB1	fungal B1 aflatoxin
AgNTs	Ag-nanotriangles
AI	artificial intelligence
AIE	aggregation-induced emission
APTES	3-aminopropyltriethoxysilane
CFU	colony forming unit
CV	cyclic voltammetry
DENV2 NS1	dengue virus type 2 NS1
FRET	Förster Resonance Energy Transfer
HIV-1	Human Immunodeficiency Virus
ISE	ion-selective electrode
LDA	linear discriminant analysis
LPS	lipopolysaccharides
LNA	locked nucleic acid
MEIS	Microfluidic Electrochemical Integrated Sensor
MNV	Murine Norovirus
s NIR	near-infrared region
PCR	polymerase chain reaction
PDMS	polydimethylsiloxane
PGs	peptidoglycans
PFU	plaque-forming unit
PPE	poly(3,4-propylenedioxythiophen-alt-3,4-ethylenedioxythiophene)
PPV	poly(phenylene vinylene)
PT	polythiophene
QCM	quartz crystal microbalance
RCA	rolling cycle amplification
SERS	surface-enhanced Raman scattering
TPE	tetraphenylethylene
UTI	urinary tract infection
Van	Vancomycin
